# New Modulators for IGF-I Activity within IGF-I Processing Products

**DOI:** 10.3389/fendo.2013.00042

**Published:** 2013-03-27

**Authors:** Becky K. Brisson, Elisabeth R. Barton

**Affiliations:** ^1^Department of Anatomy and Cell Biology, School of Dental Medicine, Pennsylvania Muscle Institute, University of PennsylvaniaPhiladelphia, PA, USA

**Keywords:** IGF-I, E-peptides, signaling, skeletal muscle, hypertrophy, glycosylation

## Abstract

Insulin-like growth factor I (IGF-I) is a key regulator of muscle development and growth. The pre-pro-peptide produced by the *Igf1* gene undergoes several post-translational processing steps to result in a secreted mature protein, which is thought to be the obligate ligand for the IGF-I receptor (IGF-IR). However, the significance of the additional forms and peptides produced from *Igf1* is not clear. For instance, the C-terminal extensions called the E-peptides that are part of pro-IGF-I, have been implicated in playing roles in cell growth, including cell proliferation and migration and muscle hypertrophy in an IGF-IR independent manner. However, the activity of these peptides has been controversial. IGF-IR independent actions suggest the existence of an E-peptide receptor, yet such a protein has not been discovered. We propose a new concept: there is no E-peptide receptor, rather the E-peptides coordinate with IGF-I to modulate activity of the IGF-IR. Growing evidence reveals that the presence of an E-peptide alters IGF-I activity, whether as part of pro-IGF-I, or as a separate peptide. In this review, we will examine the past literature on IGF-I processing and E-peptide actions in skeletal muscle, address the previous attempts to separate IGF-I and E-peptide effects, propose a new model for IGF-I/E-peptide synergy, and suggest future experiments to test if the E-peptides truly modulate IGF-I activity.

## IGF-I Regulates Skeletal Muscle Growth and Repair

Insulin-like growth factor I (IGF-I) has endocrine and autocrine/paracrine activities that regulate pre- and postnatal growth in many tissues. The main source of IGF-I is the liver (Schwander et al., 1983), which secretes IGF-I into the circulation. However, many cell types, including skeletal muscle, produce, and respond to IGF-I. IGF-I promotes growth via binding to and activating its transmembrane tyrosine kinase receptor, IGF-I Receptor (IGF-IR). Upon IGF-I binding, the IGF-IR cytoplasmic domain is autophosphorylated, which initiates multiple signaling cascades and leads to increased growth, protein synthesis, and survival.

The IGF-I pathway is an essential component of growth and repair in mature skeletal muscle. Since muscle fibers are post-mitotic, growth and regeneration rely on a stem cell-like niche of quiescent pre-muscle cells called satellite cells (Mauro, 1961). Once activated by signals for growth, overload, or injury, satellite cells proliferate, migrate to the region of the muscle that requires extra nuclei, and differentiate by fusing with myofibers (Florini et al., 1996). IGF-I is involved in many of these steps, such as myoblast proliferation and differentiation (Quinn et al., 1994; Quinn and Haugk, 1996). These processes are mediated through IGF-IR downstream pathways (Philippou et al., 2007). The mitogen-activated protein kinase (MAPK) pathway, which includes extracellular signal-regulated kinase 1 and 2 (ERK1/2), increases proliferation and migration in satellite cells and myoblasts. The phosphoinositide 3-kinase (PI3K/Akt) pathway is also stimulated, which increases differentiation and protein synthesis in mature muscle fibers (Johnson and Allen, 1990; Coolican et al., 1997; Leloup et al., 2007).

The necessity of IGF-I activity for muscle growth and repair was established through several animal models. For instance, IGF-IR inactivation in skeletal muscle leads to 10–30% lower mass (Fernandez et al., 2002; Mavalli et al., 2010) and delayed regeneration after injury (Heron-Milhavet et al., 2010). Accordingly, one can also enhance growth processes by increasing IGF-I, by infusion of recombinant IGF-I (Adams and McCue, 1998), transgenic muscle-specific over-expression (Coleman et al., 1995; Musaro et al., 2001), or viral gene delivery (Barton-Davis et al., 1998). These strategies cause hypertrophy, improve diseased muscle phenotype and function (Lynch et al., 2001; Barton et al., 2002), accelerate regeneration after injury (Rabinovsky et al., 2003; Schertzer and Lynch, 2006), and enhance hypertrophy in response to resistance training (Lee et al., 2004). Thus, there is great interest in therapeutic use of IGF-I for driving muscle growth.

## IGF-I Protein Processing

**Figure 1:**
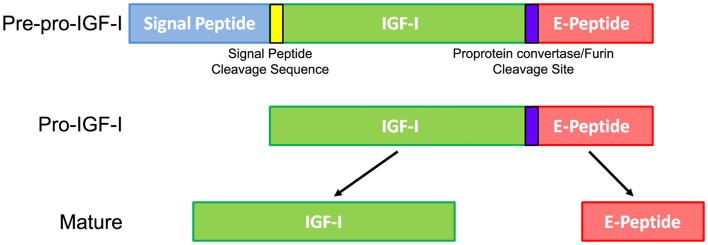
**Insulin-like growth factor I processing leading to mature IGF-I**. The *Igf1* gene is first translated into a Pre-pro-IGF-I precursor protein that includes a signal peptide, signal peptide cleavage site, IGF-I, pro-protein convertase cleavage site, and E-peptide. During translation, the signal peptide is removed from the remaining protein, now called Pro-IGF-I. Further protease cleavage separates the mature IGF-I from free E-peptide. While mature IGF-I has many accepted growth effects on a wide variety of cells and tissues, the purpose and actions of the E-peptides are relatively unknown.

The general consensus is that all IGF-I activity is mediated by mature IGF-I, but the *Igf1* gene encodes more than this protein. In the early 1980s, it was proposed that IGF-I was synthesized as a precursor protein requiring proteolysis at both the N- and C-termini to produce mature IGF-I (Jansen et al., 1983). Mature IGF-I consists of 70 amino acids (Rinderknecht and Humbel, 1978), but the full-length precursor, pre-pro-IGF-I, contains a signal peptide, mature IGF-I, and a C-terminal E-peptide extension (Figure 1). The N-terminal signal peptide is cleaved during translation in the ER, resulting in Pro-IGF-I. The E-peptide is so named because it follows the B-C-A-D domains of mature IGF-I, like the domains of insulin (Steiner, 1969). The E-peptide begins at amino acid 71, which is in a unique pentabasic prohormone cleavage motif Lys-X-X-Lys-Arg^71^-X-X-Arg-X-X-Arg^77^ (Duguay et al., 1995). Subtilisin-related proprotein convertases like furin can cleave polypeptides that include this motif, resulting in free mature IGF-I and an E-peptide (Duguay et al., 1995, 1997; Duguay, 1999). Intriguingly, uncleaved pro-IGF-I is detectable in conditioned media and *in vivo* in serum (Powell et al., 1987; Conover et al., 1989, 1993; Wilson et al., 2001; Barton et al., 2012; Durzynska et al., 2013a). To date, however, it is unclear if pro-IGF-I is bioactive or simply an inactive precursor or source for mature IGF-I and/or E-peptides.

The complexity of *Igf1* is heightened by extensive alternative splicing. The critical splicing events that determine which pro-IGF-I isoform is expressed occur at the 3′ end of the *Igf1* gene, but all isoforms retain an invariant mature IGF-I sequence. In rodents and other non-primate mammals, there are two *Igf1* isoforms called IGF-IA (IA) and IGF-IB (IB), containing E-peptides A and B (EA and EB, respectively). In humans, there are three possible isoforms: A, B, and C, where the B form is unique to humans (reviewed in Barton, 2006a). In all species, the predominant IGF-I isoform produced is IA, which is the most conserved across all species examined (Shimatsu and Rotwein, 1987; Lowe et al., 1988; Shamblott and Chen, 1993; Lund, 1998; Wallis, 2009). For an extensive discussion on IGF-I splicing, please (see Tahimic et al., 2013) in this Frontiers Research Topic.

A second facet of complexity results from glycosylation of IGF-IA. Rodent EA has two potential N-glycosylation sites, and human EA has one (Bach et al., 1990). In a recent study, we saw that IGF-I in skeletal muscle was predominantly glycosylated pro-IGF-I (Durzynska et al., 2013a), suggesting that there is a biological purpose for glycosylation on EA. Many extracellular proteins are glycosylated and interact with the extracellular matrix (ECM), and so EA glycosylation could provide IGF-I storage in the ECM for subsequent activity by cleavage (Jansen et al., 1983). However, the extent of glycosylation or cleavage may vary across different cell types. Thus, it is possible that retaining EA is a way to keep IGF-I within the muscle tissue, potentially attached to the ECM. Taken together, the conservation of alternative splicing and glycosylation suggest a physiological reason for retaining these processes. We propose that there are multiple active forms of IGF-I, and that processing provides means for altering IGF-I potency, stabilization, and/or storage.

## Searching for E-Peptide Biological Activity: A History

Since only the E-peptides differ between IGF-I isoforms, it was suggested that the E-peptides themselves had activity (Shimatsu and Rotwein, 1987). Initial studies focused on the unique human E-peptide, hEB (Siegfried et al., 1992), and found increased proliferation in human bronchial epithelial cells with hEB exposure. When a neutralizing antibody to IGF-IR was added to the proliferation assay, hEB could still induce proliferation, suggesting that hEB does not act through IGF-IR. Years later, it was discovered that hEB localizes to nucleoli (Tan et al., 2002), induces neuroblastoma cell differentiation, and increases neurite growth and ERK1/2 phosphorylation (Kuo and Chen, 2002). Recent data from our lab shows that full-length hEB increases proliferation and migration in multiple human cell lines (Durzynska et al., 2013b). Thus, the first E-peptide studied did have biological activity presumably independent from IGF-I actions.

Additional evidence for active E-peptides originates from studies of rainbow trout, *Oncorhynchus mykiss*, where there are four E-peptides (Ea-1, Ea-2, Ea-3, and Ea-4) homologous to mammalian EA (Shamblott and Chen, 1993). Recombinant Ea-2, -3, and -4 increase proliferation in mouse fibroblasts, transformed human embryonic kidney cells, and human mammary gland tumor cells (Tian et al., 1999), supporting cross-species conservation of activity. In addition, Ea-2 and -4 diminish cancer colony formation, enhanced cell attachment, and reduce invasive activity similar to hEB (Chen et al., 2002). These studies added to the hypothesis that there is biological significance for the E-peptides in the *Igf1* gene.

Focus on the IB/hIC isoform, specifically in skeletal muscle, began in the late 1990s, when the Goldspink laboratory found that while resting muscle expressed only IA, stretched rabbit muscles undergoing hypertrophy expressed both IA and IB (Yang et al., 1996). The group postulated that the IB isoform was responsible for the stretch induced hypertrophy, and renamed it Mechano Growth Factor (MGF) to distinguish it from the liver forms of IGF-I. After muscle injury, there was a transient increase in IB/MGF expression which occurred just prior to the activation of satellite cells (Hill and Goldspink, 2003; Hill et al., 2003). The temporal expression of IB/MGF led to an attractive hypothesis that this isoform, or more specifically the EB/hEC peptide, was responsible for activating satellite cells, leading to muscle hypertrophy. Consistent with this, the group showed the IB/MGF response diminished with age, where there is limited satellite cell activation (Owino et al., 2001; Hameed et al., 2003). Given the interest of the lay muscle community in factors that can drive muscle growth, MGF took on a life of its own, even though there was limited substantial, peer-reviewed evidence for its potency. Ultimately, several groups have demonstrated that synthetic MGF (corresponding to the last 24 or 25 amino acids of the IB/hIC E-peptide) drives proliferation and migration of satellite cells and myoblasts, but at the expense of differentiation (Yang and Goldspink, 2002; Mills et al., 2007b; Philippou et al., 2009; Kandalla et al., 2011).

Although much effort has focused on MGF/EB/hEC activity, curiously few studies have explored the activity of the unique human EB, and virtually no studies have examined EA. In some ways EA would be expected to have a more substantial biological function, because it is more highly expressed than the other isoforms (Lowe et al., 1988), and because the sequence is highly conserved in many species, whereas other splice forms diverge even within primates (Wallis, 2009). This suggests that if any conserved function can be ascribed to the E-peptides, the EA peptide is the most likely candidate.

## What is the Ideal Form of IGF-I for Muscle Growth: With or Without an E-Peptide?

With the ongoing dispute over E-peptide significance, we wondered if they were dispensable for IGF-I actions. In other words, could IGF-I function in the absence of the E-peptide? Using viral mediated gene transfer into mouse muscle, we expressed two rodent IGF-I isoforms (IA and IB) as well as an IGF-I lacking either E-peptide (mature IGF-I). Interestingly, both IA and IB caused more hypertrophy compared to mature IGF-I only, implying that the E-peptide is necessary and important for proper IGF-I secretion, IGF-IR activation, and/or downstream signaling (Barton et al., 2010). These findings are consistent with the phenotypes in two transgenic mouse models with muscle-specific IGF-I expression. The first mouse model expressed IGF-IA, and the mice exhibited robust skeletal muscle hypertrophy (Musaro et al., 2001). However, a mouse expressing mature IGF-I only displayed no hypertrophy (Shavlakadze et al., 2006). Together, these results suggest E-peptides are required for IGF-I to cause hypertrophy, or that their presence enhances IGF-I effects in muscle.

To understand how this enhancement might occur, we expressed fluorescently tagged constructs in myoblast cell culture to test E-peptide effects on IGF-I secretion and uptake (Pfeffer et al., 2009). We found equivalent secretion of mature IGF-I, pro-IGF-IA, and pro-IGF-IB, supporting that E-peptides were not necessary for this process. However, E-peptides enhanced the internalization of IGF-I, a step required for IGF-IR activation. Thus, IGF-I activity, including internalization and skeletal muscle hypertrophy, is improved in the presence of the E-peptides.

## E-Peptide Activity: Dependent or Independent of IGF-I?

Although the field has tried to document IGF-I *independent* activity of the E-peptides, very early on it was proposed that the E-peptides possessed IGF-I *dependent* activity, altering IGF-I secretion, or its association with IGF binding proteins or the receptors (Lowe et al., 1988; Goldspink, 1997). However, since the *Igf1* gene encodes one E-peptide for every mature IGF-I, it is difficult to discriminate IGF-I and E-peptide effects, especially in light of the fact that most of the published functions of the E-peptides are similar to IGF-I actions. Certainly, the most logical receptor for E-peptides to modulate is IGF-IR. Alternatively, if the E-peptides do have independent activity, they likely signal through their own E-peptide receptor. Many studies have blocked IGF-IR with neutralizing antibodies, and demonstrate retention of hEB and synthetic MGF activity for heightened proliferation and migration of different cell lines (Siegfried et al., 1992; Yang and Goldspink, 2002; Mills et al., 2007a,b; Philippou et al., 2009; Stavropoulou et al., 2009). These results suggest that E-peptides have activity independent of IGF-IR signaling. However, no E-peptide receptors have been found, nor have any E-peptide binding partners been discovered. While IGF-I independent activity may occur, it does not exclude that IGF-I and the E-peptides could interact, converging on the IGF-IR pathway. Evidence from our lab supports this possibility, where E-peptides modulate IGF-I signaling and uptake (Barton, 2006b; Pfeffer et al., 2009). Further, the E-peptides stimulate the same signaling pathways (MAPK) found downstream of IGF-IR and many other receptors (Kuo and Chen, 2002; Philippou et al., 2009; Stavropoulou et al., 2009). Additionally, E-peptide actions on proliferation and differentiation resemble those of IGF-I itself (Kandalla et al., 2011).

## E-Peptides Augment IGF-I Activity

**Figure 2:**
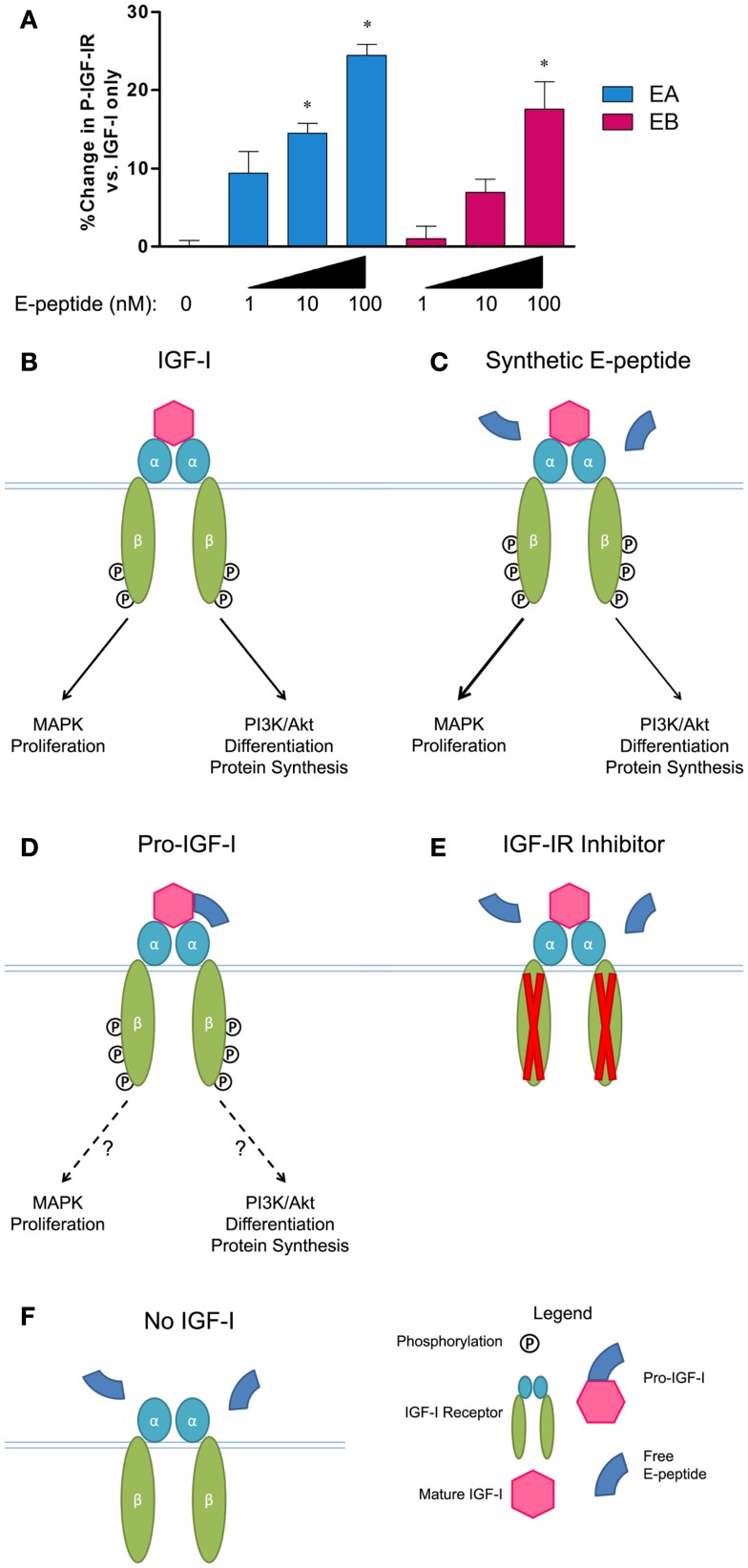
**E-peptides affect IGF-IR signaling**. **(A)** A Kinase Receptor Activation Assay (KIRA) to specifically measure IGF-IR phosphorylation was used. IGF-I at 2 nM was tested in addition to increasing amounts of synthetic E-peptides. Both EA and EB showed a dose-dependent augmentation of IGF-IR phosphorylation compared to IGF-I alone. Data taken from Brisson and Barton (2012). **(B)** Normal conditions when IGF-I is added to myoblasts, activating IGF-IR, and leading to increases in MAPK and PI3K/Akt signaling. **(C)** When IGF-I is present, synthetic E-peptides increase receptor activation and tune downstream signaling toward the MAPK pathway. **(D)** Pro-IGF-I leads to more IGF-IR phosphorylation than mature IGF-I alone, but the consequences on downstream signaling are unknown. **(E)** E-peptide MAPK activation is inhibited via NVP AEW541, an IGF-IR inhibitor. **(F)** E-peptide IGF-IR augmentation requires IGF-I.

Recently, we have directly tested the effects of E-peptides on IGF-IR signaling (Brisson and Barton, 2012) in order to clarify the mechanisms underlying E-peptide activity. Counter to previous studies, we found that both EA and EB increased ERK1/2 phosphorylation in myoblast culture, but not when IGF-IR is inhibited. To determine if the E-peptides activate IGF-IR directly, we tested if treatment of E-peptides alone, or in combination with IGF-I, could induce IGF-IR phosphorylation. We found that the E-peptides do not directly activate IGF-IR alone, but enhance the ability of IGF-I to activate IGF-IR (Figures 2A–C). Further, when myoblasts are treated with IGF-I and E-peptides, the E-peptides increase phospho-ERK1/2, but not phospho-Akt. We propose that E-peptides modulate IGF-IR signaling by enhancing the MAPK pathway, but not the PI3K/Akt pathway, therefore tuning IGF-IR downstream signaling.

Complementary to these results, we found that pro-IGF-I can drive IGF-IR phosphorylation similarly, or to even a greater extent than mature IGF-I (Figure 2D) (Durzynska et al., 2013a), suggesting that IGF-I is more potent at receptor activation when still attached to EA. Similar to simultaneous EA and IGF-I exposure causing a ∼25% increase in receptor phosphorylation compared to IGF-I alone (Figures 2A,C), when pro-IA and mature IGF-I are compared in IGF-IR activation assays, pro-IA is 20–40% more potent that mature IGF-I (Durzynska et al., 2013a). In contrast, glycosylation of pro-IGF-I appears to impair IGF-IR activation. Structurally, the C-terminal E-peptide extension faces away from the ligand binding site on IGF-IR, suggesting that it does not interfere with mature IGF-I/IGF-IR association (Vajdos et al., 2001). In fact, when a polyethylene glycol (PEG) group is attached to IGF-I where the E-peptide would be, the large PEG group still allows IGF-I/IGF-IR binding (Metzger et al., 2011). Although it is unclear if PEG-IGF-I mimics pro-IGF-I or glycosylated-pro-IGF-I, we speculate that the small E-peptide protruding from IGF-I when it is bound to IGF-IR can still have activity.

Unlike the previous studies showing IGF-I independent activity of the E-peptides, our current findings demonstrate a requirement for IGF-I presence in E-peptide activity. First, in myoblasts when the IGF-IR receptor kinase activity is inhibited via pharmacologic inhibition (Figure 2E), the E-peptides can no longer increase MAPK signaling, showing that E-peptide MAPK stimulation is IGF-IR dependent. Second, when no IGF-I is present, the E-peptides fail to increase receptor activation (Figure 2F).

Can IGF-I dependent and independent activity of the E-peptides co-exist? While our results counter the existence of an IGF-I independent pathway for the E-peptides, we cannot exclude the possibility. The experimental strategies employed differ across studies, including different cell lines, methods to increase E-peptides, and reagents to examine IGF-IR dependence. For instance, blockade of IGF-IR signaling by neutralizing antibodies can lead to receptor internalization and degradation (Chow et al., 1998; Hailey et al., 2002), and this may confound the interpretation of E-peptide effects, since we know that E-peptides affect receptor internalization and localization (Pfeffer et al., 2009; Brisson and Barton, 2012).

New data from outside our lab support the idea that the E-peptides modulate IGF-I activity, but through a different mechanism. The E-peptides contain a high percentage of basic amino acids, and are thus highly charged. One group tested if the E-peptides had an affinity for charged surfaces, and discovered that the E-peptides could tether pro-IGF-I to the ECM. Hence, the E-peptides can store IGF-I locally in tissues. The ECM tethering modifies not only IGF-I location, but also its bioavailability (Hede et al., 2012). This mechanism could explain why pro-IGF-I enhances growth *in vivo* more than mature IGF-I (Barton et al., 2010), but it cannot explain the E-peptides effect on IGF-IR activation, as those experiments were performed with free E-peptides that were not attached to IGF-I. Consequently, the E-peptides may be modulating IGF-I localization and activity through multiple mechanisms.

## Future Directions

Previous studies examining the activity of the E-peptides may have attempted to eliminate IGF-IR activity, but no study to our knowledge has tried to remove IGF-I itself, to directly test if the E-peptides require IGF-I for activity. We have addressed this issue by examining IGF-IR activation with and without exogenous IGF-I. However, even in experiments where IGF-I is not specifically added, there is likely IGF-I present in the cell media, as most cells produce and secrete IGF-I. Thus, the E-peptides must be tested in a truly “IGF-I-free” environment, to confirm that E-peptide activity is IGF-I dependent. Cells or mouse models that do not express IGF-I could be used in future experiments to address this issue.

The free E-peptides or the E-peptides included in pro-IGF-I isoforms could affect the on/off rates of IGF-I/IGF-IR binding, which in turn could affect receptor downstream signaling. In fact, the result that MAPK is stimulated while PI3K/Akt is not after E-peptide treatment suggests that the there is a change in the kinetics of receptor-ligand interaction, which has a greater impact on activation of the PI3K/Akt pathway than on the MAPK pathway (Denley et al., 2005). The PI3K/Akt pathway is more sensitive to changes in IGF-I/IGF-IR binding, and this could explain why the E-peptides modulate IGF-IR, but favor MAPK to PI3K/Akt signaling.

The largest unanswered question is how do the E-peptides augment IGF-IR activation? It could be that the E-peptides bind to or recruit other proteins that can modulate tyrosine kinases. Even though there is evidence in the literature that the E-peptides do not bind to the same binding site as IGF-I (Siegfried et al., 1992; Kuo and Chen, 2003), they could potentially still bind IGF-IR, but not in the IGF-I binding pocket. This issue, as well as whether or not the E-peptides have the same effects *in vivo* as they do in myoblast cell culture, are further directions that must be addressed.

## Conflict of Interest Statement

The authors declare that the research was conducted in the absence of any commercial or financial relationships that could be construed as a potential conflict of interest.
